# Pelvic Retroperitoneal Solitary Fibrous Tumor: Two Cases Including Doege-Potter Syndrome

**DOI:** 10.7759/cureus.110061

**Published:** 2026-06-01

**Authors:** Emma C Gómez, Sandra Diaz Casas, Patricia López Correa, Carlos Vega, Laura Arce, Natalia Lasprilla, Erika J Benito, Mauricio Garcia Mora

**Affiliations:** 1 Department of Breast and Soft Tissue Surgery, Instituto Nacional de Cancerología, Bogotá, COL; 2 Department of Oncological Pathology, Instituto Nacional de Cancerología, Bogotá, COL; 3 Department of Oncological Surgery, Instituto Nacional de Cancerología, Bogotá, COL; 4 Department of Surgery, Universidad Militar Nueva Granada, Bogotá, COL

**Keywords:** early diagnosis, pelvic tumor, rare tumors, retroperitoneal tumor, solitary fibrous tumor (sft)

## Abstract

Solitary fibrous tumor (SFT) is a rare mesenchymal lesion that typically exhibits no aggressive behavior and has low metastatic potential. While it is commonly described in the pleura and thorax, its occurrence in the retroperitoneal pelvic cavity is uncommon. We report two cases of pelvic retroperitoneal SFT treated at the Instituto Nacional de Cancerología in Colombia. The first case involved a 76-year-old man with a pelvic mass that developed over one year. Computed tomography (CT) showed a retroperitoneal tumor near the prostate and a suspicious pulmonary nodule. Biopsy confirmed high-risk SFT with signal transducer and activator of transcription 6 (STAT6) positivity and a Ki-67 index of 40%. The patient underwent extensive surgical resection with clear margins, during which an incidental prostatic adenocarcinoma was identified. At 12 months of follow-up, no evidence of recurrence was observed. The second case involved a 35-year-old man with severe hypoglycemia due to Doege-Potter syndrome secondary to pelvic SFT. Imaging demonstrated a large pelvic retroperitoneal mass causing obstructive uropathy, requiring bilateral nephrostomies. Biopsy confirmed high-risk SFT with STAT6 positivity and a Ki-67 index of 40%. The patient underwent extensive surgical resection complicated by massive intraoperative bleeding, requiring a two-stage procedure. Pathology revealed margins in contact; however, the postoperative course was favorable. At six months of follow-up, the patient remained free of hypoglycemic episodes and without radiological progression. SFT remains a rare condition, especially when located in the retroperitoneal pelvic region. Both cases highlight the clinicopathological variability of the disease and emphasize that early diagnostic suspicion and multidisciplinary management are crucial to optimizing treatment outcomes.

## Introduction

Solitary fibrous tumor (SFT) is a rare mesenchymal lesion that occurs in approximately 2.8 per 100,000 people but has a low metastatic potential. It is primarily located in the pleura and was first described by Klemperer and Rabin in 1931 [1]. Reports in the literature indicate that it can occur at extrapleural sites in 16% of cases [2]. With a 10-year survival rate of up to 73% [3], there are no differences in presentation between men and women, with a peak incidence in adults aged 40-70 years [4]. It can be associated with paraneoplastic syndromes, including Doege-Potter syndrome, which is characterized by the production of insulin-like growth factor 2 (IGF2) by tumor cells [5]. Diagnosing SFT is challenging; contrast-enhanced computed tomography (CT) and magnetic resonance imaging (MRI) are the diagnostic imaging techniques of choice to assess tumor extent, and pathologic evaluation with complete immunohistochemical analysis is essential for diagnostic certainty [6]. The nuclear expression of signal transducer and activator of transcription 6 (STAT6), reflecting the *NAB2-STAT6* fusion, is considered pathognomonic for SFT.

We report two cases of pelvic SFT, highlighting the importance of early diagnostic suspicion and a multidisciplinary approach. Written informed consent was obtained from both patients for publication.

## Case presentation

Case 1

A 76-year-old male patient was admitted to the Instituto Nacional de Cancerología in Colombia with a gradually enlarging pelvic mass of approximately one-year duration, without urinary obstruction or bowel abnormalities. The patient had a history of heavy smoking. MRI showed a large retroperitoneal mass (171 × 108 × 101 mm) near the prostate, displacing the bladder and rectum without evident infiltration of either organ. A chest CT scan revealed a suspicious pulmonary nodule, considered a possible second primary lung tumor. A Tru-Cut biopsy demonstrated a mesenchymal neoplasm consistent with solitary fibrous tumor (SFT), with strong nuclear STAT6 positivity (Figure 1).

**Figure 1:**
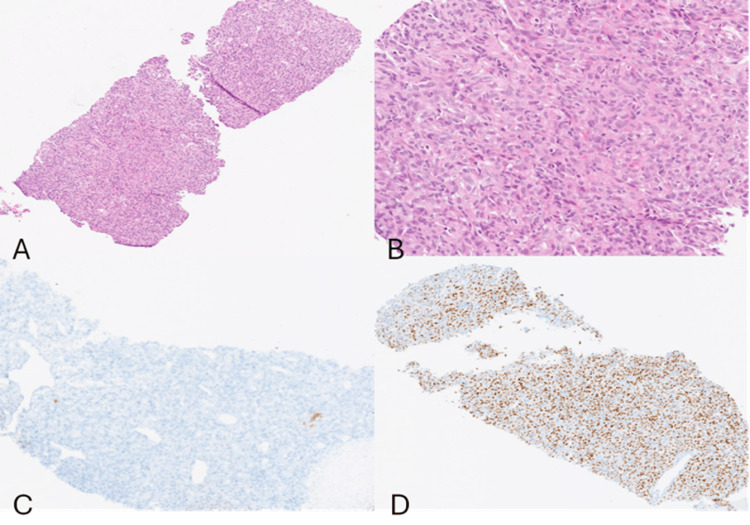
Case 1: biopsy. (A-B) Hematoxylin-eosin. A lesion is observed consisting of small spindle cells with scant cytoplasm, displaying moderate pleomorphism, arranged in a sclerosed stroma with some hyalinized vascular walls. (C) CD34: negative. (D) STAT6: strong nuclear positivity.

The patient underwent extensive surgical resection through laparotomy. Intraoperatively, the tumor was found adherent to the prostate, bladder, and urethra, with a urethral laceration that was repaired during the procedure. Histopathological examination confirmed a high-risk SFT according to the Demicco risk stratification model (score 7), measuring 18 × 13.5 × 10 cm, with <10% necrosis, a Ki-67 index of 40%, and negative surgical margins (Figure 2). An incidental focus of prostatic acinar adenocarcinoma (Gleason score of 3 + 3, measuring 0.2 cm) was also identified. The patient remains under follow-up by oncology and thoracic surgery services to determine the management of the pulmonary nodule and the possible role of adjuvant radiotherapy.

**Figure 2:**
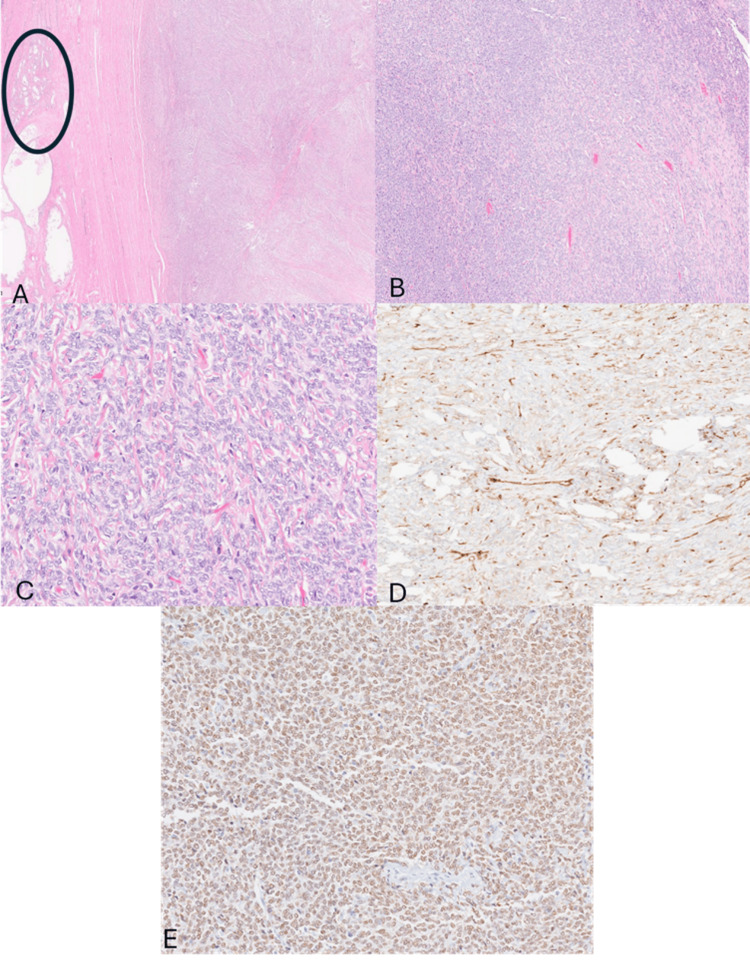
(A-C). Hematoxylin-eosin. Pelvic tumor and prostate resection product. (A) A well-circumscribed spindle cell lesion is observed, with prostatic glandular tissue attached externally. A focus of acinar adenocarcinoma is identified in the prostatic tissue (circled). (B and C) Lesion made up of small spindle cells with scant cytoplasm, showing moderate pleomorphism, arranged in a sclerosed stroma that contains some hyalinized vascular walls. (D) CD34: positive. (E) STAT6: nuclear positivity.

Case 2

A 35-year-old male patient was admitted because of recurrent episodes of severe hypoglycemia associated with altered consciousness. Preoperative glucose values during hypoglycemic episodes were 24, 24, and 30 mg/dL. Imaging studies identified a 16 cm pelvic retroperitoneal mass causing the compression of the bladder, rectum, and ureters, with associated bilateral hydronephrosis (Figure 3). Thoracoabdominal CT showed no evidence of metastatic disease. Although serum IGF2 levels were not measured, the hypoglycemia was retrospectively attributed to Doege-Potter syndrome secondary to tumor-related IGF2 secretion. A Tru-Cut biopsy confirmed the diagnosis of SFT through positivity for STAT6 and CD34 (Figure 4). Due to obstructive uropathy, the patient required urinary diversion with bilateral nephrostomies before surgery. During the operation, a hypervascular pelvic tumor densely adherent to urogenital and vascular structures was identified. Extensive resection was performed with the preservation of the ureters and bladder; however, the ligation of the right internal iliac artery was required (Figure 5).

**Figure 3:**
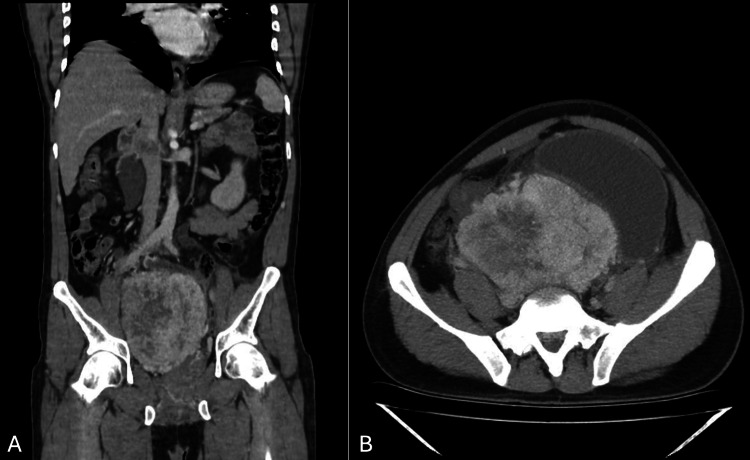
Case 2: pelvic SFT CT image. (A) Coronal contrast-enhanced CT showing a well-defined and large mass. (B) Axial CT image demonstrating a mass occupying the pelvic cavity. SFT, solitary fibrous tumor; CT, computed tomography

**Figure 4:**
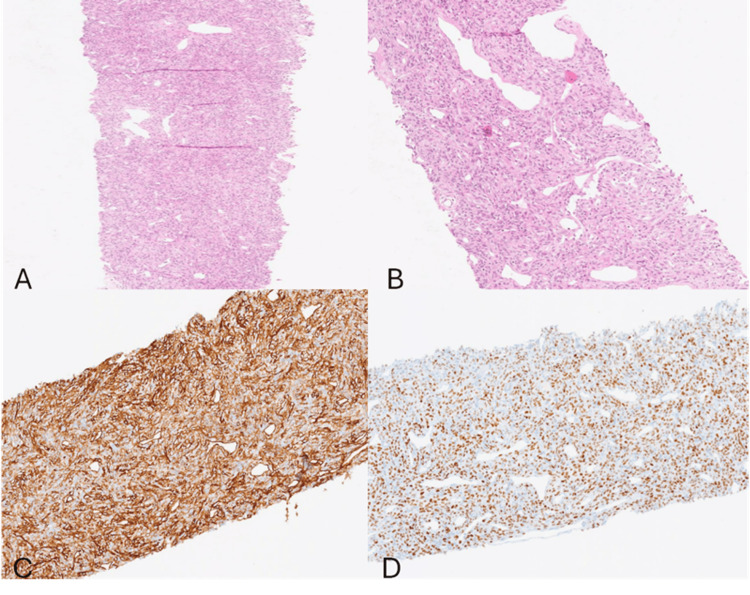
(A and B) Hematoxylin-eosin. A mesenchymal lesion is observed, composed of uniform spindle cells with elongated nuclei and scant cytoplasm, without atypia, with branched blood vessels and perivascular hyalinization forming a hemangiopericytoma-like pattern. (C) CD34: positive in the neoplastic cells. (D) STAT6: strong nuclear positivity.

**Figure 5:**
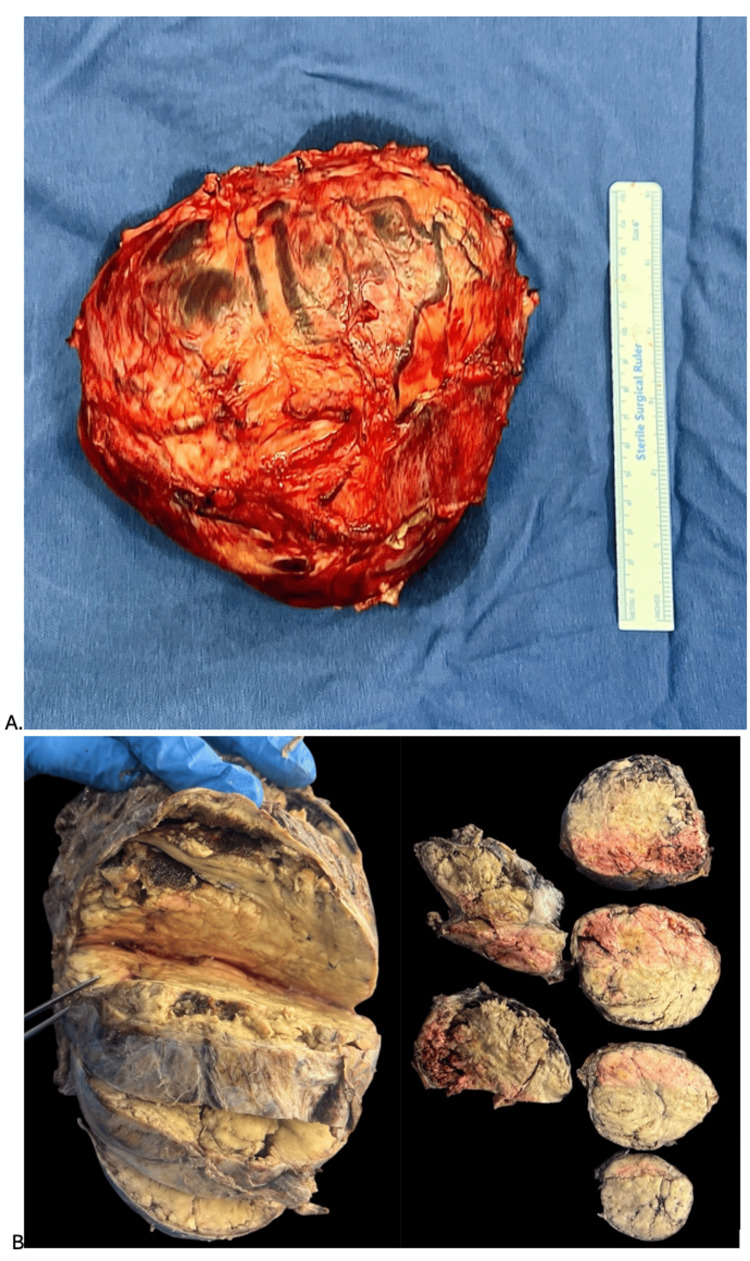
(A) Hypervascularized mass measuring 16 × 20 cm with well-defined borders. (B) Macroscopic sections of the surgical specimen.

The procedure was complicated by massive intraoperative bleeding of approximately 3,000 mL, requiring transfusion of eight units of packed red blood cells, seven units of fresh frozen plasma, and one platelet apheresis unit. Pelvic packing was performed, and a second-stage procedure was completed 48 hours later for pelvic unpacking and definitive abdominal closure. Postoperative laboratory tests demonstrated leukocytes of 8,900/mm³, hemoglobin of 11 g/dL, platelets of 496,000/mm³, and glucose of 118 mg/dL. The patient recovered without major complications. Final pathology confirmed high-risk SFT with approximately 10% necrosis, a Ki-67 proliferation index of 40%, and surgical margins in contact with the lesion (Figure 6). The patient was discharged in stable condition and remains under postoperative oncologic follow-up, without the recurrence of hypoglycemic episodes.

**Figure 6:**
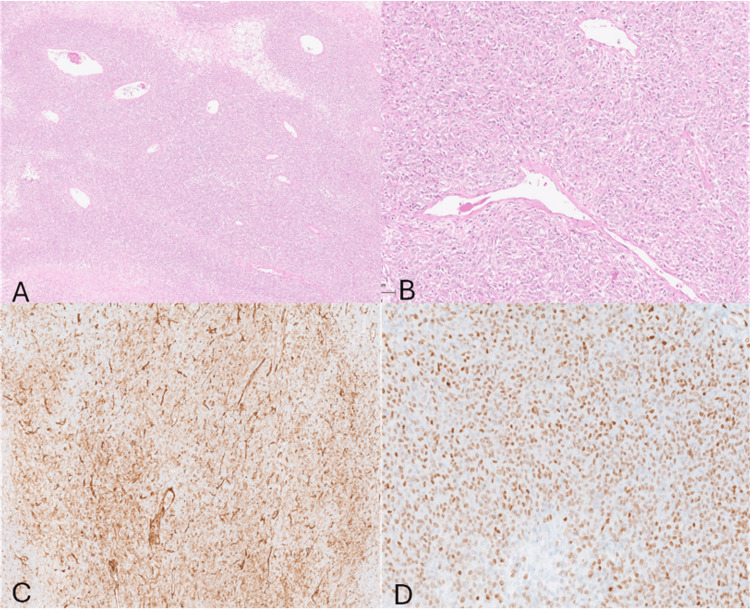
Case 2: resection product of a retroperitoneal tumor. (A and B) Hematoxylin-eosin. A mesenchymal lesion is observed, made up of uniform spindle cells with elongated nuclei and minimal cytoplasm, without atypia. It features branched blood vessels and perivascular hyalinization, forming a hemangiopericytoma. (C) CD34: positive in the neoplastic cells. (D) STAT6: strong nuclear positivity.

## Discussion

Retroperitoneal sarcomas are rare and heterogeneous pathologies, with at least 100 different histological subtypes recognized. The four most common in the retroperitoneum are liposarcoma (both dedifferentiated and well-differentiated, at 40% and 24%, respectively), leiomyosarcoma (at 20%), and solitary fibrous tumor (at 5%) [7]. The last type presents a diagnostic and treatment challenge. Although our report is limited by the small number of cases and its single-institution design, these cases provide valuable clinical and surgical insights into the presentation and multidisciplinary management of pelvic retroperitoneal solitary fibrous tumors.

Diagnosis

The presentation of SFT is usually asymptomatic or causes symptoms due to mass effect, including progressive chronic abdominal or lumbar pain radiating to a limb, weight loss, neuromotor deficits or sensory changes, anemia, lower limb edema, abdominal distension, and, in less common cases, symptoms caused by hypoglycemia [8,9].

Computed tomography is an imaging technique that aids in the diagnostic process. These lesions are characterized by a well-defined, hyperdense mass with heterogeneous post-contrast enhancement due to hypervascularization on CT scans, which is rarely associated with calcifications or necrosis [8,9].

On the other hand, abdominal MRI shows an iso-hypointense lesion on T1 and heterogeneous on T2, with intense enhancement after gadolinium. There are no pathognomonic radiological signs, but the images help define the lesion’s extent and the surgical plan [8,9].

Histopathology

SFT generally does not show aggressive behavior, and the WHO 2020 classification places it in the group of fibroblastic/myofibroblastic lesions with intermediate potential [10]. Histologically, it is characterized by a dilated, branching, and hyalinized vasculature with a classic “staghorn” or hemangiopericytoma-like vascular pattern [11]. These tumors typically present with low mitotic activity and mild nuclear pleomorphism [5]. However, cases with necrosis, infiltrative growth pattern, large tumor size, and elevated mitotic count tend to behave more aggressively [5] and may have a metastatic potential of up to 30% [10-12]. The French Federation of Cancer Centers Sarcoma Group (FNCLCC) classification is not routinely used for this tumor type; therefore, specific risk stratification models for SFT have been developed. One of the most widely used is the Demicco risk stratification model, which incorporates age, necrosis, mitotic count, and tumor size to classify patients into metastatic risk groups. In our report, both patients were classified as high risk for metastasis, with scores greater than 6.

Confirmatory diagnosis is established through immunohistochemistry. Signal transducer and activator of transcription 6 (STAT6), a specific marker for detecting the *NAB2-STAT6* fusion gene [13], is directly involved in tumor formation and exhibits high sensitivity and specificity in tumor cells [14]. The pericentric inversion on chromosome 12 (inv(12)(q13q13)), which fuses the *NAB2* and *STAT6* genes, is the pathognomonic and defining molecular feature of SFT, including those in pelvic locations. This alteration results in a fusion protein that transforms the repressive activity of *NAB2* into an activating function, enhancing the expression of genes such as *EGR1* and promoting tumorigenesis [15,16].

Associations with other entities

Paraneoplastic syndromes are defined as various metabolic or clinical changes that indicate systemic involvement. These syndromes are rare and are seldom mentioned in the literature as a presentation of sarcomas [17]. In the second case described here, a paraneoplastic syndrome developed, characterized by non-insulin-mediated hypoglycemia (non-islet cell tumor hypoglycemia, NICTH), also known as Doege-Potter syndrome, which results from IGF2 production in tumor cells [18]. Other clinical and physiological conditions associated with dedifferentiated retroperitoneal sarcomas include hyperglycemia, thrombocytopenia, fever, thrombocytosis, and leukemoid reaction [17].

Paraneoplastic syndromes in retroperitoneal SFT are rare and infrequent, with limited specific reports in the medical literature. In the case series by Takizawa et al., hypoglycemia was reported as the main symptom of paraneoplastic syndrome in 11.5% of patients [19]. Meanwhile, a case series by Bratu et al. evaluated a cohort of patients with retroperitoneal mesenchymal tumors and reported paraneoplastic syndromes in 9.37% of patients; however, not all patients in this publication were diagnosed with SFT [20].

Other rare paraneoplastic syndromes associated with SFT include idiopathic hypertrophic osteoarthropathy, known as Pierre-Marie-Bamberger syndrome, reported in less than 10% of cases, characterized by pain and the thickening of long bones due to increased vascular factors; digital clubbing, linked to the secretion of mediators such as vascular endothelial growth factor (VEGF) and platelet-derived growth factor (PDGF); and even paraneoplastic hypercalcemia syndrome [15,21].

Surgical approach

Complete surgical resection with adequate margins is the ideal treatment for these sarcomas [20]. The MD Anderson Cancer Center published a study in which combined therapy with surgical resection and radiotherapy was performed, achieving 100% local control, 95% overall survival, and 92% distant disease-free survival at five years of follow-up in patients with SFT in the head and neck, extremities, and trunk (represented by lesions in the retroperitoneum, pelvis, and axilla) [22]. This suggests the potential use of neoadjuvant and adjuvant radiotherapy for this histological sarcoma type [23,24].

From a surgical perspective, in both cases described, patients underwent an extensive resection of large retroperitoneal pelvic masses via a midline laparotomy, with the careful dissection of urogenital and vascular structures. In the first case, the complete removal of a 25 × 20 × 16 cm tumor, firmly attached to the prostate, bladder, and seminal vesicles, was achieved, along with the partial resection of the bladder wall and an intraoperative urethral laceration that required primary anastomosis and bladder descent, without major complications. In the second case, the surgery was particularly complex due to a 17 × 12 × 11 cm hypervascular mass involving the right internal iliac artery, which had to be ligated because of massive hemorrhage (3,000 mL). This was managed with the transfusion of multiple blood products, pelvic packing, and a second, delayed surgical revision. In both scenarios, treatment was targeted to achieve free margins, as recommended in the literature.

New therapies

With the advent of new therapies, especially targeted therapies, the first prospective multicenter clinical trial was conducted by Martin-Broto et al., which evaluated the antitumor activity and safety of the receptor tyrosine kinase (RTK) inhibitor pazopanib in SFT, reporting an overall response rate of 58% according to Choi criteria [24]. This offers an alternative for managing this pathology when criteria for inoperability or disease progression are present.

This was a single-arm, phase 2 trial that enrolled adult patients (≥18 years) diagnosed with a confirmed metastatic or unresectable typical SFT of any location, who had progressed within the previous six months, according to Choi criteria or response evaluation criteria in solid tumors (RECIST), and had an Eastern Cooperative Oncology Group (ECOG) performance status of 0-2, at 11 tertiary hospitals in Italy, France, and Spain. After defining the eligibility criteria, 31 patients were enrolled. During an 18-month follow-up (interquartile range {IQR}: 14-34), 18 (58%) had a partial response, 12 (39%) had stable disease, and one (3%) showed progressive disease according to Choi criteria and central review. The overall response rate based on Choi criteria was 58% (95% CI = 34-69) [24]. The above suggests that this RTK inhibitor drug could be considered as a first-line treatment for advanced SFT.

## Conclusions

SFT is a rare mesenchymal neoplasm encompassing several clinicopathological subgroups. Its retroperitoneal pelvic location presents a diagnostic challenge because of the nonspecific clinical manifestations and the absence of pathognomonic radiological findings. The early identification of associated signs and symptoms, together with the integration of clinicopathological characteristics, immunohistochemistry, and molecular testing, is essential for accurate diagnosis. For any hypervascular pelvic spindle cell tumor, SFT should be included in the differential diagnosis, and STAT6 immunohistochemistry should be performed. A multidisciplinary approach involving surgical oncology, pathology, clinical oncology, and radiation oncology enables comprehensive management focused on complete surgical resection and surveillance for recurrence or metastasis, particularly in high-risk tumors. Long-term follow-up is warranted given the metastatic potential of up to 30% reported in high-risk SFT.
